# Cardiovascular Risk Factors in Childhood and Adulthood and Cardiovascular Disease in Middle Age

**DOI:** 10.1001/jamanetworkopen.2024.18148

**Published:** 2024-06-24

**Authors:** Noora Kartiosuo, Olli T. Raitakari, Markus Juonala, Jorma S. A. Viikari, Alan R. Sinaiko, Alison J. Venn, David R. Jacobs, Elaine M. Urbina, Jessica G. Woo, Julia Steinberger, Lydia A. Bazzano, Stephen R. Daniels, Costan G. Magnussen, Kazem Rahimi, Terence Dwyer

**Affiliations:** 1 Research Centre of Applied and Preventive Cardiovascular Medicine, University of Turku, Turku, Finland; 2 Centre for Population Health Research, University of Turku and Turku University Hospital, Turku, Finland; 3 Department of Mathematics and Statistics, University of Turku, Turku, Finland; 4 Division of Medicine, Turku University Hospital, Turku, Finland; 5 Murdoch Children’s Research Institute, Melbourne, Victoria, Australia; 6 Department of Clinical Physiology and Nuclear Medicine, Turku University Hospital, Turku, Finland; 7 Department of Internal Medicine, University of Turku, Turku, Finland; 8 Department of Medicine, University of Turku, Turku, Finland; 9 Department of Pediatrics, University of Minnesota Medical School, University of Minnesota, Minneapolis; 10 Menzies Institute for Medical Research, University of Tasmania, Hobart, Tasmania, Australia; 11 Division of Epidemiology and Community Health, School of Public Health, University of Minnesota, Minneapolis; 12 Heart Institute, Cincinnati Children’s Hospital Medical Center, Cincinnati, Ohio; 13 Department of Pediatrics, University of Cincinnati College of Medicine, Cincinnati, Ohio; 14 Division of Biostatistics and Epidemiology, Cincinnati Children’s Hospital Medical Center, Cincinnati, Ohio; 15 Department of Epidemiology, Tulane University School of Public Health and Tropical Medicine, New Orleans, Louisiana; 16 University of Colorado School of Medicine, and Anschutz Medical Campus, Children’s Hospital Colorado, Aurora; 17 Baker Heart and Diabetes Institute, Melbourne, Victoria, Australia; 18 Baker Department of Cardiometabolic Health, Faculty of Medicine, Dentistry and Health Sciences, University of Melbourne, Melbourne, Victoria, Australia; 19 Alliance for Research in Exercise, Nutrition and Activity, University of South Australia, Adelaide, Australia; 20 Nuffield Department of Women’s and Reproductive Health, University of Oxford, Oxford, United Kingdom

## Abstract

**Importance:**

Recent evidence suggests that childhood levels of serum lipids, blood pressure, body mass index (BMI), and smoking contribute to adult risk of cardiovascular disease (CVD). Evidence is lacking on whether this is independent of adult risk levels.

**Objective:**

To quantify direct and indirect effects of childhood risk factors on adult CVD via adulthood risk factors using mediation analysis, and to quantify their relative importance during different life-course stages using a life-course approach.

**Design, Setting, and Participants:**

This prospective cohort study followed participants from the US, Finland, and Australia from childhood (1970s-1990s) until 2019, with data on CVD risk factors in childhood and adulthood. Longitudinal childhood and adulthood risk factors were summarized to describe BMI, lipids, and blood pressure cumulatively. Childhood and adulthood smoking were assessed with questionnaires. Data analysis was performed May 2022 to August 2023.

**Main Outcomes and Measures:**

The primary outcomes were fatal and nonfatal cardiovascular events in adulthood. Mediation analysis was used to estimate the direct and indirect effects of the childhood risk factors with CVD events, reported as incidence rate ratios (RRs) and 95% CIs.

**Results:**

A total of 10 634 participants (4506 male participants [42.4%]; mean [SD] age at childhood visit, 13.3 [3.0] years; mean [SD] age at adulthood visit, 32.3 [6.0] years) were included in the cohort. The mean (SD) age at CVD event or censoring was 49.2 (7.0) years. The median (IQR) follow-up time was 23.6 (18.7-30.2) years. Childhood risk factors, (low-density lipoprotein cholesterol [LDL-C], total cholesterol [TC], triglycerides, systolic blood pressure [SBP], smoking, BMI, and a combined score of these) were associated with CVD. BMI (direct effect for incidence RR per 1 SD unit, 1.18; 95% CI, 1.05-1.34) and LDL-C (direct effect incidence RR, 1.16; 95% CI, 1.01-1.34) in particular were found to play an important role via direct pathways, whereas the indirect effects were larger for TC, triglycerides, SBP, and the combined score. Childhood smoking only affected CVD via adulthood smoking. Life-course models confirmed that for the risk of CVD, childhood BMI plays nearly as important role as adulthood BMI, whereas for the other risk factors and the combined score, adulthood was the more important period.

**Conclusions and Relevance:**

In this cohort study of 10 634 participants, childhood risk factors were found to be associated both directly and indirectly to adult CVD, with the largest direct effect seen for BMI and LDL-C. These findings suggest that intervention for childhood risk factors, in particular BMI, is warranted to reduce incidence of adult CVD as it cannot be fully mitigated by risk factor management in adulthood.

## Key Points

**Question:**

Are childhood cardiovascular risk factors associated with cardiovascular disease (CVD) in middle age independently of adulthood risk factors?

**Findings:**

This cohort study of 10 634 participants who were followed from childhood to adulthood found that childhood levels of low-density lipoprotein cholesterol, total cholesterol, triglycerides, systolic blood pressure, smoking, body mass index, and a combined score of these were associated with CVD in adulthood, partially independent of adulthood levels of these factors.

**Meaning:**

These findings suggest that intervening in the risk factors associated with CVD, especially body mass index, in childhood should be emphasized to reduce the risk of CVD later in life.

## Teaser

This cohort study examines the associations of childhood risk factors, including low-density lipoprotein cholesterol, total cholesterol, triglycerides, systolic blood pressure, smoking, body mass index, and a combined score of these, with cardiovascular disease (CVD) in adulthood.

## Introduction

The prevention of cardiovascular disease (CVD) through reduction of established risk factors has largely focused on interventions commencing in midadulthood. However, the underlying pathology, atherosclerosis, can start in childhood, and its early development depends on the same risk factors.^1,2^ Thus, even greater reduction in CVD incidence might be achieved by interventions commencing in childhood. Conceptually, risk could accumulate with duration of exposure to the risk factors, similarly to the effects of smoking with lung cancer.^3,4^

Growing evidence from cohort studies^1,5,6,7,8,9,10,11^ has linked childhood CVD risk factors to adult preclinical atherosclerotic vascular phenotypes. Furthermore, mendelian randomization studies have found that carrying low-density lipoprotein cholesterol (LDL-C)–lowering variants is associated with doubly reduced risk of CVD, compared with randomized trials producing a similar cholesterol-lowering outcome during adulthood only.^12^ Similarly, using cholesterol-lowering agents early in life for familial hypercholesterolemia reduces adulthood CVD risk more than starting use of such agents later in life.^13^

Despite these grounds for suspecting that childhood risk factors are associated with adult CVD, there is limited population-level evidence to support this hypothesis. In prospective data from the International Childhood Cardiovascular Cohort (i3C) Consortium, we reported that childhood risk factors were associated with incidence of adult CVD.^14^ This was observed for individual childhood risk factors, their combined risk score, and the change in risk score between childhood and adulthood, suggesting that both childhood risk factor levels and the path to risk in adulthood are important.

However, the previous research did not fully address how individual childhood risk factors may be associated with CVD risk independently of adulthood risk factor levels, and how much is owing to indirect effects via tracking of CVD risk factors from childhood to adulthood,^15,16^ nor have the importance of different CVD risk factors at particular stages during the life course been investigated to determine whether certain life periods act as more important periods of exposure. Obtaining insights into the contribution of CVD risk factors at different life stages may inform early-life strategies for CVD prevention. The current article presents an analysis of the direct and indirect effects of childhood risk factors with CVD in adulthood and an examination of the relative contribution of risk factors at different stages along the life course.

## Methods

### Study Design and Sample

The i3C consortium consists of 7 longitudinal cohort studies from Australia, Finland, and the US. The studies commenced between 1970s and 1990s, when the participants were aged 3 to 19 years. Each cohort has since collected prospective data on cardiovascular risk factors, described in detail previously.^14,17,18^

Our analyses included a subset of the original 38 589 participants who had at least 1 measurement in both childhood and adulthood and follow-up for CVD events after age 25 years (eFigure 1 in Supplement 1). Each study was approved by the associated university or hospital district ethical authorities. In childhood, oral participant consent and written parental permission were obtained. Written informed participant consent was obtained in adulthood. Oral consent under waiver of documentation of consent was obtained for the recent follow-up questionnaire. This report follows the Strengthening the Reporting of Observational Studies in Epidemiology (STROBE) reporting guidelines for cohort studies.

### Responses

Information about adulthood CVD events was collected using death registries, a national health database (Finland), or an i3C self-report questionnaire adjudicated using hospital records (Australia and US). The CVD events of interest included myocardial infarction, stroke, transient ischemic attack, ischemic heart failure, angina, peripheral artery disease, abdominal aortic aneurysm, carotid intervention, and coronary revascularization; heart failure due to nonatherosclerotic diseases was excluded. In our main analyses, we explore fatal and nonfatal CVD events combined, and fatal events separately in the additional analyses. The non-CVD deaths were discarded from our analyses.

### Risk Factor Measures and Covariates

The risk factors investigated include body mass index (BMI; calculated as weight in kilograms divided by height in meters squared), systolic blood pressure (SBP), total cholesterol (TC), LDL-C, log_e_-transformed triglycerides (logTG), and tobacco smoking.^14^ Height, weight, and blood pressure were measured at the clinic visits.^19^ Plasma or serum cholesterol and TG levels were measured using standard methods. Smoking was assessed by questionnaires in childhood and adulthood.

We used the i3C-derived *z* scores of the risk factors as described elsewhere.^14^ In brief, the longitudinally measured risk factors were normalized to *z* scores by age and sex. We then calculated the participant-specific mean risk factors for childhood (3-19 years), early childhood (3-11 years), adolescence (12-19 years), adulthood (from age 20 years until event or censoring), and early adulthood (20-30 years) as the mean of the normalized measurements over these time spans. A combined risk score was built by calculating the mean of *z* scores of BMI, SBP, logTG, and TC and smoking, where smoking was included as 2 (smokers; high risk in *z* score units) or 0 (nonsmokers; average risk).^14^ LDL-C was not included in the risk score because of sample size and high correlation between LDL-C and TC. The risk factors and *z* scores are described in more detail in eAppendix 1 in Supplement 1.

Sex, self-reported race , cohort, mean age and year of childhood measurements, mean age of adulthood measurements (in models including adulthood), childhood socioeconomic status (SES) as maximum level of parental reported education, and adulthood SES as maximum level of own education were considered as covariates. For analyses, race data were categorized as Black, White, and other, with other including Asian, American Indian or Alaska Native, Native Hawaiian or Pacific Islander, more than one race, and unknown. Because less than 5% of participants were classified as other, we further collapsed the categories to the binary of Black and all other categories combined (which was mostly White participants) for analysis. The decision to include race in our analysis was guided by our aim to determine the association of race with our findings, considered alongside social, historical, and cultural influences, on health outcomes without implying any genetic or biological determinants. See eAppendix 1 in Supplement 1 for assessment and harmonization.

### Statistical Analysis

Data analysis was performed from May 2022 to August 2023. We used mediation analysis to estimate the direct and indirect effects of the childhood risk factors with CVD events and report incidence rate ratios (RRs) and corresponding 95% CIs. The bayesian relevant life-course model was used to quantify the life-course importance of the risk factors, and the posterior distribution means and their 95% credible intervals (CrIs) are presented.^20^ The data were multiply imputed to correct for nonresponse bias using PROC MI in SAS statistical software version 9.4 (SAS Institute) with 10 replications. We have described our multistage imputation procedure on imputing the risk factors, CVD event status, time-to-event and covariates in detail previously.^14^

#### Mediation Analysis

**Figure 1.**
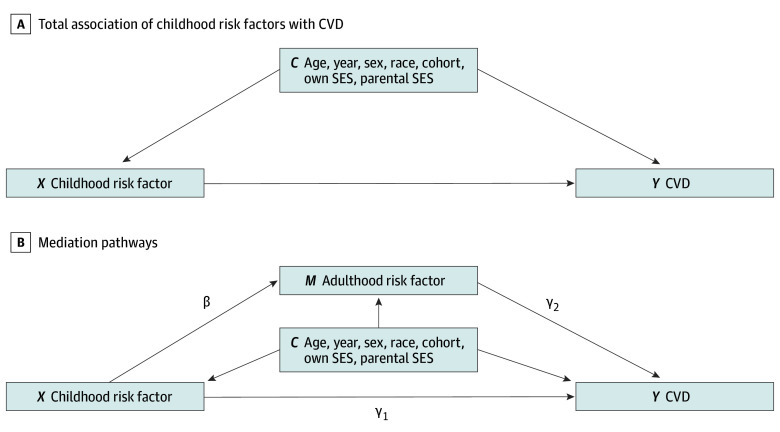
Directed Acyclic Graph of the Mediation Analysis Framework Panel A shows the total effect of childhood risk factors with cardiovascular disease (CVD), adjusted for covariates. Panel B shows the mediation pathways (ie, the direct effect γ_1_ and the β and γ_2_ parameters related to the indirect effects). In addition to the variables shown in the figure, logarithm of the time between beginning of the follow-up and the event or end of the follow-up was included in the Poisson models as an offset term. SES indicates socioeconomic status.

We used Poisson regression to obtain the RRs of CVD events, treating logarithm of the follow-up time (ie, time from age 25 years until CVD event or end of the follow-up) as an offset. The hypothesized mediation pathways are visualized as a directed acyclic graph in Figure 1. The total effect refers to the association of childhood risk factors on CVD without accounting for the adulthood risk factor levels considered as mediators (Figure 1A), whereas the direct effect (arrow γ_1_ in Figure 1B) corresponds to the association of childhood risk factors on CVD, adjusted for the adulthood risk factors. The indirect effect is composed of 2 parts and is calculated using the product-of-coefficients approach. The first part describes tracking (ie, the effect of childhood risk factor with the adulthood risk factor) (arrow β in Figure 1B), estimated using linear regression for the continuous risk factors and logistic regression for the binary smoking.^21^ The second part describes the effect of adulthood risk factor with CVD, adjusted for the childhood risk factor levels (arrow γ_2_ in Figure 1B). We present more detailed formulas for the indirect effects in eAppendix 2 in Supplement 1. Total, direct, and indirect effects were estimated separately for each imputation and pooled using Rubin rule.

#### Bayesian Relevant Life-Course Model

To understand the relative importance of childhood risk factor levels at different stages during the life course and to identify the life-course hypotheses supported by our data, we estimated the relative contributions of each risk factor to the CVD event during different life stages using the bayesian relevant life-course model.^20^ The method and our application are described in more detail in eAppendix 2 in Supplement 1. Briefly, we fitted a model explaining CVD with the risk factor levels at the life-course periods of interest and estimated importance of risk factor at life-course periods as relative weights. The weights obtained are used to interpret the life-course hypotheses that the data might support, including accumulation (all periods are equally important; that is, they have equal weights), sensitive period (one period has larger weight than the other periods), and critical period (only one period during the life-course is important while the others have a small weight).

On the basis of these weights (constrained to take value between 0 and 1 and sum to 1), relevant life-course exposure was built as a weighted sum of the risk factors at different life-course periods. Simultaneously, for this sum, the life-course coefficient (not constrained) δ, describing the association of the weighted sum with CVD is obtained. Of note, we here relied on a logistic model for the binary CVD event owing to the exploratory nature of these analyses. The covariates included indicator of male sex, race, indicators of geographic location (Australia, Finland, Midwest, or Bogalusa), and standardized mean year of childhood examination.

We fitted the models using 2 life-course periods (childhood, ages 3-19 years; adulthood, age 20 years until the last measurement before event or censoring) for the exposures. In addition, the models were fitted for 3 life-course periods (early childhood, ages 3-11 years; adolescence, ages 12-19 years; adulthood, age 20 years until last measurement before event or censoring) for the i3C-derived *z* score measures of continuous risk factors. Smoking and the subsequent risk score were omitted from the latter analysis due to insufficient smoking information in early childhood.

The models were fitted separately for each imputation using PROC MCMC in SAS. We report the mean weights and the mean coefficient of the life-course exposure effect and their 95% CrIs averaged over the imputations.

## Results

### Basic Characteristics

Of the 10 634 participants in the analysis sample, 4506 (42.4%) were male, 1036 (9.7%) were Black, and the remaining 9598 (90.3%) were other races. The mean (SD) age was 13.3 (3.0) years at the childhood visit and 32.3 (6.0) years at the adulthood visit (Table 1). The median (IQR) follow-up time was 23.6 (18.7-30.2) years. On average over the 10 imputations, 520.9 participants (4.9%; range, 499-544 participants; of these, 406 were observed and the rest imputed) had any fatal or nonfatal CVD event (Table 1) at the mean (SD) age of 49.2 (7.0) years. eTable 1 in Supplement 1 presents the risk factor levels in childhood and adulthood for the participants by outcome group. For the total and LDL-C, we present the levels separately for the Finnish participants, because in Finland the levels of LDL-C have changed considerably over the period of data collection.^22^ In the analyses, this is accounted for by adjusting for cohort or location of the participants; in addition, a sensitivity analysis without the Finnish participants was conducted for the mediation analyses.

**Table 1.** Basic Characteristics by Event Status Averaged Over the 10 Imputations

Characteristic	Participants, No. (%)		
	All (N = 10 634)	Nonevent (n = 10 113.1)	CVD event, fatal or nonfatal (n = 520.9)
Sex			
Female	6128 (57.6)	5900.3 (58.3)	227.7 (43.7)
Male	4506 (42.4)	4212.8 (41.7)	293.2 (56.3)
Race			
Black	1036 (9.7)	952.2 (9.4)	83.8 (16.1)
Other^a^	9598 (90.3)	9160.9 (90.6)	437.1 (83.9)
Cohort			
Bogalusa Heart Study	1742 (16.4)	1565.6 (15.5)	176.4 (33.9)
Childhood Determinants of Adult Health	2021 (19.0)	1999.8 (19.8)	21.2 (4.1)
Minnesota Childhood Cardiovascular Cohorts	859 (8.1)	845.3 (8.4)	13.7 (2.6)
Muscatine Study	2153 (20.3)	1943.4 (19.2)	209.6 (40.2)
National Heart, Lung, and Blood Institute Growth and Health Study	435 (4.3)	449.0 (4.4)	4.0 (0.8)
Princeton Lipid Research Study	557 (5.2)	514.0 (5.1)	43.0 (8.3)
Cardiovascular Risk in Young Finns Study	2849 (26.8)	2796.0 (27.7)	53.0 (10.2)
Parental education			
Less than high school	2437.5 (22.9)	2361.2 (23.4)	76.3 (14.7)
High school	3204.1 (30.1)	2920.7 (28.9)	283.4 (54.4)
Greater than high school, some college	2283.6 (21.5)	2215.2 (21.9)	68.4 (13.1)
College degree or higher	2708.8 (25.5)	2616.0 (25.9)	92.8 (17.8)
Own education			
Less than high school	429.6 (4.0)	384.1 (3.8)	45.5 (8.7)
High school	2383.6 (22.4)	2197.8 (21.7)	185.8 (35.7)
Greater than high school, some college	3638.8 (34.2)	3483.5 (34.5)	155.3 (29.8)
College degree	2712.8 (25.5)	2616.4 (25.9)	96.4 (18.5)
College degree or higher	1469.2 (13.8)	1431.3 (14.2)	37.9 (7.3)
Smoker in childhood	4077.6 (38.3)	3800.1 (37.6)	277.5 (53.3)
Smoker in adulthood	3442.0 (32.4)	3155.0 (31.2)	287.0 (55.1)
Birth year, mean (SD)	1967 (7.3)	1967 (7.2)	1961 (5.6)
Age, mean (SD), y			
Time of event or censoring	49.2 (7.0)	49.2 (6.7)	49.7 (7.0)
Childhood visits	13.3 (3.0)	13.2 (3.1)	14.2 (2.5)
Early childhood visits	10.2 (1.7)	10.2 (1.7)	10.7 (1.5)
Adolescent visits	14.9 (1.7)	14.9 (1.7)	15.2 (1.6)
Adulthood visits before event or censoring	32.3 (6.0)	32.2 (6.0)	32.4 (6.0)

Abbreviation: CVD, cardiovascular disease.

^a^ The category other in race mostly consists of White participants, but also includes participants identifying as Asian, American Indian or Alaska Native, Native Hawaiian or Pacific Islander, more than 1 race, and unknown. eAppendix 1 in Supplement 1 describes collection and harmonization of race data in more detail.

### Mediation Analysis

**Figure 2.**
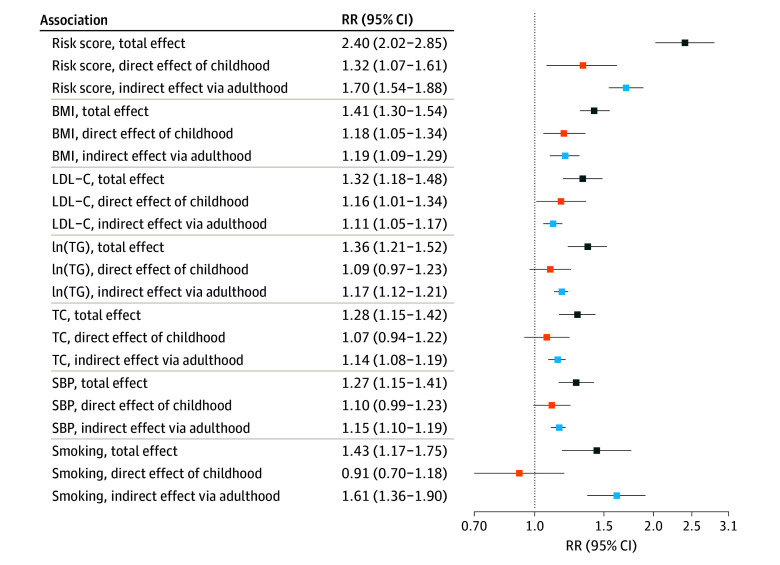
Total, Direct, and Indirect Effects of Childhood Risk Factors on Cardiovascular Events The risk factors are ordered according to the magnitude of their direct contribution. BMI indicates body mass index; LDL-C, low-density lipoprotein cholesterol, ln(TG), log_e_-transformed triglycerides; RR, rate ratio; SBP, systolic blood pressure; TC, total cholesterol.

Figure 2 shows the total, direct, and indirect effects for each risk factor. The 2 pathways composing the indirect effects are presented in eTable 2 in Supplement 1. Each childhood risk factor contributed to CVD, corresponding to what we reported previously (total effects).^14^ Of the continuous risk factors investigated, BMI clearly contributed both directly (RR, 1.18; 95% CI, 1.05-1.34) and indirectly (RR, 1.19; 95% CI, 1.09-1.29) (Figure 2). For LDL-C, both direct (RR, 1.16; 95% CI, 1.01-1.34) and indirect (RR, 1.11; 95% CI, 1.05-1.17) pathways were important, whereas for the other continuous risk factors, including logTG (RR, 1.17; 95% CI, 1.12-1.21), TC (RR, 1.14; 95% CI, 1.08-1.19), and SBP (RR, 1.15; 95% CI, 1.10-1.19), the indirect pathways contributed more than the direct pathways. For smoking, only indirect effects were positive (RR, 1.61; 95% CI, 1.36-1.90). The combined risk score was more important than any of the risk factors alone (direct effect RR, 1.32; 95% CI, 1.07-1.61; and indirect effect RR, 1.70; 95% CI, 1.54-1.88).

In mediation analysis for fatal CVD events, the total, direct, and indirect effects remained similar to the main analysis (eTable 3 in Supplement 1). The sex-stratified mediation analyses indicated that indirect effects of smoking were larger in female than in male participants, whereas for male participants, the direct effect of SBP was substantially larger than for female participants (eTable 4 in Supplement 1). The participants with multiple childhood measurements had larger direct and indirect effects compared with those with only 1 visit (eTable 5 in Supplement 1). As BMI may be considered a causal antecedent of the lipids and blood pressure, we present mediation analysis results adjusted for childhood and adulthood BMI *z* score measures (eFigure 2 and eTable 6 in Supplement 1).^23,24^ In the BMI-adjusted analyses, the total effects of SBP, TC, logTC, and LDL-C were slightly smaller. When treating the non-CVD events as nonevents, the results remained rather similar (eTable 7 in Supplement 1). For total and LDL-C, the mediation analysis results without the Finnish participants remained similar to those reported here (eTable 8 in Supplement 1).

### Relative Contributions of Childhood Risk Factors at Different Life-Course Periods

In the 2-period life-course models (Table 2) using the childhood and adulthood measures, the relative weight allocated for childhood BMI by the life-course model (0.44; 95% CrI, 0.17-0.68) was not very far below that of adulthood (0.56; 95% CrI, 0.32-0.83), implying that in terms of developing CVD, BMI in childhood plays nearly as important a role as in adulthood. The weights for LDL-C were 0.33 (95% CrI, 0.08-0.55) for childhood and 0.67 (95% CrI, 0.45-0.92) for adulthood. Corresponding to the mediation analysis results, the importance of childhood was slightly lower for SBP, TC, and logTG (approximately 0.20 for each), indicating that adulthood (importance approximately 0.80 for each) is more important period of exposure. For smoking, the adulthood importance was even larger (0.89; 95% CrI, 0.74-1.00) compared with childhood (0.11; 95% CrI, <0.01-0.26). For the risk score, the importance was 0.24 (95% CrI, 0.11-0.36) for childhood and 0.76 (95% CrI, 0.64-0.89) for adulthood, indicating that childhood risk score is of some importance for development of CVD, whereas adulthood acts as the more important period. Corresponding to the mediation analysis results, the combined risk score has the highest life-course coefficient, 1.54 (95% CrI, 1.34-1.74), whereas the life-course associations of individual risk factors were smaller.

**Table 2.** Posterior Distribution for the Relative Weights of the Risk Factors in Childhood and Adulthood

Risk factor	Relative weight, mean (95% credible interval)		Life-course coefficient, mean (95% credible interval)
	Childhood (age 3-19 y)	Adulthood (age 20 y to event or censoring)	
Score^a^	0.24 (0.11-0.36)	0.76 (0.64-0.89)	1.54 (1.34-1.74)
Smoking	0.11 (<0.01-0.26)	0.89 (0.74-1.00)	0.89 (0.79-1.08)
Log_e_-transformed triglycerides	0.18 (0.02-0.32)	0.81 (0.68-0.98)	0.62 (0.50-0.73)
Systolic blood pressure	0.17 (0.02-0.32)	0.83 (0.68-0.98)	0.52 (0.41-0.63)
Body mass index	0.44 (0.17-0.68)	0.56 (0.32-0.83)	0.46 (0.36-0.55)
Low-density lipoprotein cholesterol	0.33 (0.08-0.55)	0.67 (0.45-0.92)	0.41 (0.30-0.52)
Total cholesterol	0.17 (<0.01-0.36)	0.83 (0.64-1.00)	0.40 (0.30-0.51)

^a^ The score includes body mass index, systolic blood pressure, total cholesterol, log-transformed triglycerides, and smoking. The weights describe the relative importance of the risk factors at each life stage, whereas the life-course coefficient describes the association of the weighted sum of the periods on cardiovascular disease on a logit-scale. The numbers reported are posterior distribution means (average of highest posterior density interval) averaged over the 10 imputations.

The second division of the life-course into early childhood, adolescence, and adulthood (Table 3) suggests that importance of BMI is larger in adulthood, although both early childhood (relative weight, 0.18; 95% CrI, <0.01-0.40) and adolescence (relative weight, 0.31; 95% CrI, 0.01-0.58) are also periods of importance. Corresponding to the 2-period models, LDL-C seems to play an important role from early life onward, with relative weights of 0.12 (95% CrI, <0.01-0.30) for early childhood, 0.26 (95% CrI, 0.02-0.50) for adolescence, and 0.62 (95% CrI, 0.40-0.85) for adulthood. For the other risk factors, adulthood is the most important period (all relative weights >0.75 for adulthood and <0.20 for early childhood and adolescence). Of note, to some extent, the weight of adulthood in the 3-period models may stem from the length of adulthood period (from age 20 years until last measurement before censoring or event), whereas the 2 first periods are shorter.

**Table 3.** Posterior Distribution for the Relative Weights of the Risk Factors in Early Childhood, Adolescence, and Adulthood^a^

Risk factor	Relative weight, mean (95% credible interval)			Life-course coefficient, mean (95% credible interval)
	Early childhood (age 3-11 y)	Adolescence (age 12-19 y)	Adulthood (age 20 y to event or censoring)	
Body mass index	0.18 (<0.01-0.40)	0.31 (0.01-0.58)	0.51 (0.27-0.76)	0.46 (0.37-0.56)
Low-density lipoprotein cholesterol	0.12 (<0.01-0.30)	0.26 (0.02-0.50)	0.62 (0.40-0.85)	0.41 (0.31-0.52)
Log_e_ transformed triglycerides	0.11 (<0.01-0.23)	0.10 (<0.01-0.22)	0.79 (0.64-0.93)	0.63 (0.51-0.74)
Systolic blood pressure	0.11 (<0.01-0.24)	0.10 (<0.01-0.23)	0.79 (0.65-0.93)	0.53 (0.42-0.64)
Total cholesterol	0.09 (<0.01-0.23)	0.16 (0.01-0.35)	0.75 (0.55-0.95)	0.41 (0.31-0.52)

^a^ The weights describe the relative importance of the risk factors at each life stage, while the life-course coefficient describes the association of the weighted sum of the periods with cardiovascular disease on a logit-scale. The numbers reported are posterior distribution means (average of highest posterior density interval) averaged over the 10 imputations. Because of data availability at these different life-course periods, the sample sizes per imputation in these analyses varied between 6751 and 7148.

## Discussion

In this cohort study, we found that childhood risk factors were associated with adult CVD events via both direct and indirect pathways. BMI and LDL-C in particular were found to play an important role directly. For TC, TG, SBP, and smoking, the indirect effects were more important. The estimates for the life-course importance of different risk factors at each life stage confirmed and elaborated the mediation analysis findings, indicating that for the risk of developing CVD, childhood BMI played nearly as important a role as adulthood BMI, supporting the accumulation hypothesis. Both early childhood and adolescence BMI appeared to have some importance.

Also, LDL-C was important, especially in adolescence, whereas adulthood remained the most important period. The interpretation of these results in life-course terms is that the risk begins to materially accumulate already in childhood, with adulthood risk factor levels having the greater impact. For other risk factors, childhood levels had some degree of importance, but adulthood was a substantially more important period, supporting an adult sensitive period hypothesis.

Childhood risk factors are thus associated with risk of an adult CVD event as independent direct contributors and acting through adulthood risk factor levels, similarly to the well-established cumulative life-course effect of smoking with lung cancer.^3,4^ If this biological construct is applied to risk factors for CVD, given that childhood occupies approximately one-third of the lifespan of our participants, our life-course results may reasonably be inferred as supporting this model of pathogenesis.

Our findings allow inferring which interventions to reduce CVD risk factors in childhood might be priorities in CVD prevention programs. For BMI and LDL-C in particular, the addition of early prevention and childhood interventions to public health programs to reduce the CVD burden is supported. Although this is not so obvious for those biological risk factors that appeared more important in adulthood, the contribution of these childhood risk factors to the later adult risk factor levels also makes a compelling case for their inclusion in CVD prevention programs. Even though the effects of smoking were mostly via adulthood, our results highlight the opportunity presented in childhood and adolescence to prevent lifelong smoking, because smoking is a modifiable behavior and the majority of adult smokers start in adolescence.^25^

These findings clearly show that higher risk factors levels in childhood are associated with future CVD via both direct and indirect pathways. The results are based on individuals who have lived in the period where programs for risk factor reduction in adulthood, such as sophisticated antihypertensive agents and statins, have been available. Yet, still they have retained a much higher risk for adult CVD than their peers who had lower risk factor levels in childhood. Once risk factors are established at a high level in childhood, reducing them effectively is difficult.^26,27^ Although efforts to reduce risk factors in adulthood remain important, efforts to achieve optimal success in preventing both the direct and indirect effects we observed may need to commence in childhood.^28,29,30^ The development of public strategy that determines the desirable balance of lifestyle and clinical interventions at each life stage is now a priority. Future studies should prioritize collecting more comprehensive data on social determinants of health than we have been able to (eg, access to health care and healthy environments) to better inform the design and implementation of effective and targeted interventions.

### Limitations and Strengths

This study has limitations that should be mentioned. Our analyses concern a subpopulation of the participants previously investigated.^14^ The potential loss-to-follow-up bias in ascertaining the nonfatal cardiovascular was addressed by multiple imputation. In addition, we estimated the mediated effects for only fatal events, which were ascertained from almost all participants. Reassuringly, these results support similar interpretations as the main analyses. Furthermore, the recruitment of the cohorts was not sufficiently powered to investigate racial differences or disparities in social determinants of health. The cohorts were from high-income countries and majority of the participants were White.

The strengths of this study include unique longitudinal data with detailed life-course measurements on CVD risk factors and follow-up data on deaths on nearly the full cohort. Mediation analysis and life-course results provided similar insights on the role of risk factors in childhood, especially BMI, on CVD later in life, strengthening our conclusions.

## Conclusions

In this cohort study of 10 634 participants, childhood risk factors were found to contribute to adult CVD both directly and indirectly, via adulthood risk factor levels, with the largest direct effects seen for BMI and LDL-C. These findings suggest that increased emphasis on early risk factor prevention in childhood is justified.

## Notes

Supplement 1. **eAppendix 1.** Analysis sample, risk factor measures and covariates **eFigure 1.** Flow chart of the analysis sample **eAppendix 2.** Statistical methods **eTable 1.** The Z-scored risk factors by event status (mean (SD)) averaged over the 10 imputations **eTable 2.** The path-specific coefficients for the indirect effects **eTable 3.** Risk ratios for the total, direct and indirect effects of childhood risk factors on the fatal cardiovascular events **eTable 4.** Risk ratios for the total, direct and indirect effects of childhood risk factors on the fatal and non-fatal cardiovascular events stratified by sex **eTable 5.** Risk ratios for the total, direct and indirect effects of childhood risk factors on the fatal and non-fatal cardiovascular events stratified by number of visits in childhood **eFigure 2.** Hypothesised causal pathways of childhood SBP and lipids on CVDs, when BMI is considered antecedent of the risk factors **eTable 6.** Risk ratios for the total, direct and indirect effects for the biological risk factors when adjusting for the BMI measurements **eTable 7.** Risk ratios for the total, direct and indirect effects treating the non-CVD deaths as nonevents **eTable 8.** Risk ratios for the total, direct and indirect effects for total and LDL-C without Finnish participants
eReferences

Supplement 2.
Data Sharing Statement
