# Supplementary material for: Cardiovascular Risk Factors in Childhood and Adulthood and Cardiovascular Disease in Middle Age
Source: JAMA Netw Open. 2024 Jun 24;7(6):e2418148. doi: 10.1001/jamanetworkopen.2024.18148 (PMC11197443; doi:10.1001/jamanetworkopen.2024.18148)
Supplement: Supplement 1. — eAppendix 1. Analysis sample, risk factor measures and covariates eFigure 1. Flow chart of the analysis sample eAppendix 2. Statistical methods eTable 1. The Z-scored risk factors by event status (mean (SD)) averaged over the 10 imputations eTable 2. The path-specific coefficients for the indirect effects eTable 3. Risk ratios for the total, direct and indirect effects of childhood risk factors on the fatal cardiovascular events eTable 4. Risk ratios for the total, direct and indirect effects of childhood risk factors on the fatal and non-fatal cardiovascular events stratified by sex eTable 5. Risk ratios for the total, direct and indirect effects of childhood risk factors on the fatal and non-fatal cardiovascular events stratified by number of visits in childhood eFigure 2. Hypothesised causal pathways of childhood SBP and lipids on CVDs, when BMI is considered antecedent of the risk factors eTable 6. Risk ratios for the total, direct and indirect effects for the biological risk factors when adjusting for the BMI measurements eTable 7. Risk ratios for the total, direct and indirect effects treating the non-CVD deaths as nonevents eTable 8. Risk ratios for the total, direct and indirect effects for total and LDL-C without Finnish participants eReferences [file jamanetwopen-e2418148-s001.pdf]

Supplemental Online Content

Kartiosuo N, Raitakari OT, Juonala M, et al. Cardiovascular risk factors in childhood and adulthood to cardiovascular disease in middle age. *JAMA Netw Open*. 2024;7(6):e2418148. doi:10.1001/jamanetworkopen.2024.18148

eAppendix 1. Analysis sample, risk factor measures and covariates
eFigure 1. Flow chart of the analysis sample
eAppendix 2. Statistical methods
eTable 1. The Z-scored risk factors by event status (mean (SD)) averaged over the 10 imputations
eTable 2. The path-specific coefficients for the indirect effects
eTable 3. Risk ratios for the total, direct and indirect effects of childhood risk factors on the fatal cardiovascular events
eTable 4. Risk ratios for the total, direct and indirect effects of childhood risk factors on the fatal and non-fatal cardiovascular events stratified by sex
eTable 5. Risk ratios for the total, direct and indirect effects of childhood risk factors on the fatal and non-fatal cardiovascular events stratified by number of visits in childhood
eFigure 2. Hypothesised causal pathways of childhood SBP and lipids on CVDs, when BMI is considered antecedent of the risk factors
eTable 6. Risk ratios for the total, direct and indirect effects for the biological risk factors when adjusting for the BMI measurements
eTable 7. Risk Ratios for the Total, Direct and Indirect Effects Treating the Non-CVD Deaths as Nonevents
eTable 8. Risk Ratios for the Total, Direct and Indirect Effects for Total and LDL-C Without Finnish Participants
eReferences

This supplemental material has been provided by the authors to give readers additional information about their work.

© 2024 Kartiosuo N et al. *JAMA Network Open.*

eAppendix 1: Analysis sample, risk factor measures and covariates

Analysis sample, loss to follow-up and missing data
We have previously presented a very detailed description of loss to follow-up of the non-fatal cardiovascular event self-report and adjudication in the full i3C analysis sample.[1] Briefly, the participants who were not located for the non-fatal event follow-up more likely to be male, Black, and of lower parental and own educational status, and they were younger. The non-located had higher smoking rates, but lower SBP, TC and combined risk scores. Some participants were located, but their self-reported event status could not be adjudicated from the medical records e.g. due to the events having occurred many years prior to the self-report follow-up and the medical records not being found, or due to participants not giving consent to obtain the medical records. The participants whose records were not adjudicated were more often males and from White/Other race group, their childhood SES was lower, and they had higher SBP and TC levels in childhood. More detailed investigation is provided in Jacobs et al 2022[1], Supplementary Material S5.

eFigure 1 presents the flow chart of our analysis sample. We here investigated a subsample of the cohort that had visits in both childhood and adulthood. Adulthood visits have taken place at different life-course stages in different cohorts, and some cohorts have not attempted to follow up all their participants in adulthood. In our analysis sample, participants are included if, in addition to childhood measurement, they have a measurement taken at or after at 20 and prior to any cardiovascular event; in addition, follow-up status on CVD events after the age 25 (i.e., start of the follow-up) was required. Participants with only questionnaire data in adulthood were also excluded.

To correct for non-response bias and fully utilise the existing data, the analysis data set was multiply imputed using PROC MI in SAS with 10 replications. The multi-stage imputation procedure has been described in detail previously.[1] Briefly, we first imputed the risk factor levels and non-fatal events among the group who were not located for self-report events. Then, event status was separately imputed for the participants who had self-report event data but no adjudication. Lastly, for the imputed events, time-to-event was multiply imputed. The adulthood risk factor levels were only imputed for the subpopulation who were included in the subsample with adult measurements.

eFigure 1. Flow chart of the analysis sample.

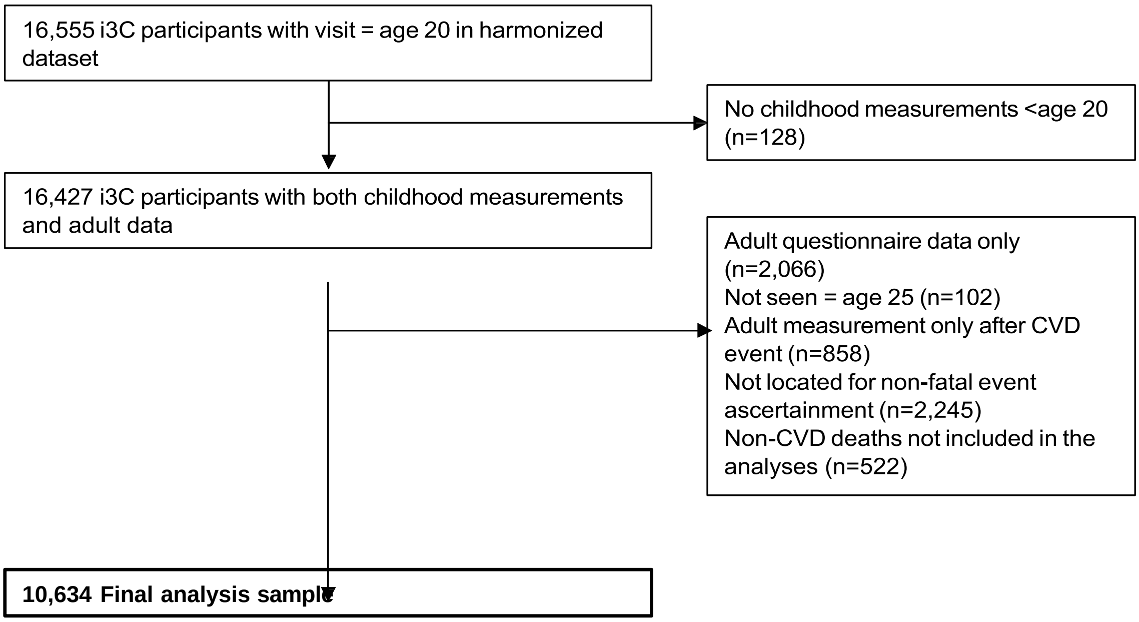

© 2024 Kartiosuo N et al. *JAMA Network Open.*

Risk factors

BMI

BMI was calculated based on the weight and height measurements as weight/(height in meters). For majority of longitudinal observations, weight and height were measured on the visit. In childhood, a subset of height and weight measurements were based on self-report in Princeton (6 %) and YFS (2 %). Also in the adulthood visits, a subset of height and weight measurements were based on self-report (Height: CDAH 44 %, Princeton 35 %, YFS 3 %; Weight: CDAH 56 %, Princeton 33 %; YFS 3%). In addition, self-reported height and weight were assessed for other cohorts than YFS in the outcomes questionnaire.

Blood pressure

Blood pressure was calculated as an average of 1-4 measurements, depending on cohort. In NGHS, BHS, CDAH and the first two time points in YFS, blood pressure was measured using standard mercury sphygmomanometry. In Muscatine, Insulin, PHBPC, Princeton and NaKS as well as in YFS starting from year 1986 blood pressure was measured using random zero. Because there is some dispute about the best way to avoid error in the measurement of diastolic blood pressure in children we have chosen to report only the systolic blood pressure measures for both adults and children.[2]

Total cholesterol, LDL-cholesterol and triglycerides

Plasma or serum cholesterol and triglycerides levels were measured using standard methods. LDL-C was estimated using HDL-C assayed after precipitation and the Friedewald's equation for the subset of participants whose HDL-C was measured.[3]

Smoking

The harmonisation of the binary youth smoking has been described in more detail previously.[1,4] Briefly, the self-report smoking during childhood was harmonised across the cohorts so that minimal smoking during adolescence or childhood was coded as non-smoking. Also, those participants who reported being former smokers were considered as having smoked in childhood. In addition, data on adult recall of starting smoking between ages 15 and 19 was utilised when available.

Derivation of risk factor measures

The longitudinal data base harmonised over the cohorts to same measurement units was used do derive the i3C Consortium-wide age- and sex-standardised z-score as described previously.[1] Briefly, each longitudinal measurement of the continuous biological risk factors was standardised with mean 0 and standard deviation 1 by sex and age categories (3-5, 6-8, 9-11, 12-14, 15-17, 18-19 years) to avoid confounding due to sex or measurement age. Then, these z-scored measurements were averaged for each participant over their life span for entire childhood (3-19 years) as well as childhood (3-11 years) and adolescence (12-19 years). We thus obtained a single measure of risk factor level over these periods for each individual. Similar approach was used for adulthood measures using age groups from 20-22 years to 38-40 years and 41+ years. Combined risk factor score was calculated as un unweighted average of the i3C z-scored BMI, SBP, TG and TC and dichotomous smoking variable coded as 0 for non-smokers (corresponding to average risk) and 2 for smokers (corresponding to a high risk z-score).

Harmonization of covariates

Race and ethnicity

Given that the consortium consists of multiple different cohorts across different countries, the assessment of race and ethnicity was variable across the cohorts. Information on race or ethnicity was assessed in the cohorts because race was considered to be an important demographic factor.

Race/ethnicity data has been collected longitudinally. Initially during the childhood visits, the parents of the participants reported race/ethnicity. Later on, if the participant took part to follow-up visits, the information was updated based on their self-report. Race data were harmonized based on all available data collected historically in each cohort as well as self-reported information in the outcomes follow-up survey.

All other cohorts assessed race/ethnicity of the participants but YFS (Finland). All YFS participants were assumed to be non-Hispanic White. In other cohorts, race was collected as follows:

© 2024 Kartiosuo N et al. *JAMA Network Open.*

- CDAH (Australia): Race was classified based on parent country of origin and language spoken at home self-reported by the participant in childhood, defaulting to non-Hispanic White in the absence of this information.
- US cohorts: Race was generally self-reported by parents during childhood or by participants during adolescence or adulthood. Non-standard race categories were re-coded to standard categories (e.g., Oriental classified as Asian), but mixed race was frequently not captured (e.g., "select one" rather than "select all"). Cohort-specific practices included:
  - Bogalusa Heart Study: Participants self-identified as 'White American' or 'African American'.
  - NGHS: Required participants to have race-concordant families (parents and child were self-reported all White or all Black).
  - Muscatine Study: Did not collect race data during childhood, and in those with adult follow-up, race and ethnicity were captured using a single question (race/ethnicity). Those missing self-reported data were assumed to be non-Hispanic White.
  - Princeton: In childhood, questions were asked about place of birth, race and "origin or descent" which captured ethnicity information. At the adult follow-up, ethnicity data were not captured, and race was assessed as "White", "Black" and "Other".
  - Minnesota: In childhood, race and ethnicity were captured in a single question (race/ethnicity), with responses of 'White', 'Black', 'Native American', 'Hispanic' and 'Mixed'.

In the Outcomes follow-up survey[5], race data across cohorts was aimed to harmonize over the cohorts by by collecting a broader and more standardized range of self-report categories (White, Black/African American, Asian, American Indian/Alaska Native, Native Hawaiian/Pacific Islander, and Other (specify)) and allowing multiple selections. 'More than One Race' was assigned to participants selecting multiple race categories. Reponses specifying 'Other' were reviewed and race and ethnicity categories were updated as possible; those indicating only Hispanic ethnicity were assigned "Unknown" for race if additional information was not available.

For analyses, race data were categorized into 'Black', 'White', and 'Other', with 'Other' including Asian, American Indian/Alaska Native, Native Hawaiian/Pacific Islander, More than One Race, and Unknown. Due to less than 5% of participants being classified as "Other", we further collapsed the categories to 'Black' and 'non-Black' for analysis. Hispanic ethnicity was missing in 70% of participants, and of those with data, 98% were non-Hispanic, so ethnicity was not included in analysis. The decision to include race in our analysis was guided by our aim to determine the impact of race on our associations, considered alongside social, historical, and cultural influences, on health outcomes without implying any genetic or biological determinants.

Childhood socio-economic status

Parental education levels used as a proxy for childhood socio-economic status. The education levels were obtained from questionnaires. The reported education levels were harmonised across the cohorts into less than high school (1); high school graduate or equivalent (2); some college and more than high school (3); and college degree or more (4).

Educational level 1 (less than high school) includes in Finnish participants those parents who did not finish from primary school or who finished primary school but did not graduate from high school or vocational institute of the equal level. In Australian participants, all parents whose reported education level was at most year 11 or equivalent were categorised into level 1. In the US cohorts, parents who had reported that their maximum education level was at most 11 years or 10-12 years, no high school or some high school, elementary school or junior high were categorised into level 1. Parental education level 2 (high school graduate or equivalent) includes graduating from high school or equivalent or having 12 years of education. The parents were categorised into education level 3 (some college, more than high school) if they had post-high school vocational training or vocational, technical or merch school (Finland), trade/apprenticeship or certificate/diploma education (Australia), or some post-high school education, college studies without graduating, AA degree, trade degree, vocational training or 13-15 years of education (US). Lastly, the parents were categorised into eduction level 4 (college degree or more) if they reported to have a BSc, MSc or PhD (Finland), university degree or higher university degree (Australia), college degree, graduate or post-graduate studies, bachelor's degree, or 16-20 years of education.

If the parents had reported their education level multiple times during the participants' childhood, the highest education level was used. In the YFS, information on parental education level was also collected when the participants were adults, and the information was only used to augment the missing values of childhood socio-economic status. The maximum education level of both parents was used in the analyses.

© 2024 Kartiosuo N et al. *JAMA Network Open.*

Adulthood socio-economic status

Own education level based on questionnaires was used as a proxy for adulthood socio-economic status. The education levels were harmonised across the cohorts into five categories: less than high school (1); high school graduate or equivalent (2); some college studies or more than high school (3); university degree (4); or higher university degree (5). The maximum education level across the questionnaires was used.

Education level 1 (less than high school) includes the participants who had no more than basic schooling (Finland), had no schooling, primary school or at most year 11 or equivalent (Australia), and those who reported their having education level less than high school graduation or less that 12th grade. Education level 2 (high school graduate or equivalent) includes participants who went to high school or had a vocational education equivalent to high school education (Finland), year 12 or equivalent (Australia), and graduating HS or GED or 12th grade (US). Education level 3 (some college studies or more than high school) was defined as post-high school vocational institute, university of applied studies or some university studies without degree (Finland), trade/apprenticeship or certificate/diploma education (Australia), and some college, 2-year Associate's degree, vocational or technical education, trade or business school, 1-3 years of college or junior/community college, Technical certificate or AA degree (US). The participants were categorised into education level 4 (university degree) if they had a bachelor's or master's degree (Finland), university degree (Australia), college, or university degree or some post-college education (US). Lastly, education level 5 (higher university degree) includes licenciate or PhD (Finland), higher university degree (Australia), completed postgraduate degree, Master's degree, Doctorate, professional degree (such as MD), or more than 5 years of college or junior/community college (US).

© 2024 Kartiosuo N et al. *JAMA Network Open.*

eAppendix 2. Statistical methods

B.1 Mediation analysis

To assess the effect of the childhood risk factors via its effect on the adulthood risk factors as well as independently of them, we used mediation analysis and quantified the *direct* and *indirect* effects of the childhood risk factors on the CVD events.[6,7] The hypothesised mediation pathways are visualised as a directed acyclic graph (DAG) in Figure 1 of the main text. Denote the response (CVD events) as Y, the childhood risk factor levels as X, the adulthood risk factor levels as M and the set of covariates (birth year, sex, race, cohort and own and parental SES) as $\boldsymbol{C}$. In practice, the total direct and indirect effects are estimated by fitting three separate models. The models were as follows:

$$(1)\ \log(E(Y|X,\boldsymbol{C})) = \alpha_0 + \alpha_1 X + \boldsymbol{\alpha'_2 C} + \ln(\text{time})$$
$$(2)\ \log(E(Y \mid X, M, \boldsymbol{C})) = \gamma_0 + \gamma_1 X + \gamma_2 M + \boldsymbol{\gamma'_3 C} + \ln(\text{time})$$
$$(3a)\ E(M|X,\boldsymbol{C}) = \beta_0 + \beta_1 X + \boldsymbol{\beta'_2 C}$$
$$(3b)\text{logit}(p(M=1)) = \beta_0 + \beta_1 X + \boldsymbol{\beta'_2 C}$$

Here, models (1) and (2) model the incidence rate ratios of CVD events using the Poisson regression approach, treating the logarithm of the follow-up time (time from age 25 until occurrence of CVD event or end of the follow-up) as an offset term (terms denoted as ln(time) in models 1 and 2).[8] Model (3a) is a general linear model used for continuous mediators whereas model (3b) is a logistic regression model, used for the binary mediator (smoking). Parameter α_1 in Model (1) is the total effect, corresponding to the effect of childhood risk factor (*X*) on CVD (*Y*) without accounting for the adulthood risk factor levels (*M*). Parameter $\gamma_1$in Model (2) is the direct effect, corresponding to the effect of childhood risk factors on CVD, adjusted for the adulthood risk factors (the effect of *X* on *Y* when adjusting for *M*, corresponding to parameter γ_1 in DAG (ii) of Figure 1).

The indirect effect was calculated based on the two coefficients, i.e., effect of childhood risk factor on the adulthood risk factor (parameter β_1 in Models (3a) or (3b)) and the effect of adulthood risk factor on CVD adjusted for the childhood risk factor levels (parameter γ_2 in Model (2)). In case of continuous mediator, the indirect effect was calculated as $\beta_1\gamma_2$ on the log-RR level and correspondingly as exp ($\beta_1\gamma_2$) on the RR level. The standard errors of the indirect effects were derived based on the Delta method.[6] Based on the framework of indirect effects under binary mediators[7], the indirect effect for smoking was calculated on the log-scale as

$$\theta_2\left(\frac{\exp(\beta_0+\beta_1 a+\beta_2' c)}{1+\exp(\beta_0+\beta_1 a+\beta_2' c)} - \frac{\exp(\beta_0+\beta_1 a^*+\beta_2' c)}{1+\exp(\beta_0+\beta_1 a^*+\beta_2' c)}\right),$$

where the levels a and a^* were chosen as 1 and 0, respectively and c corresponds to the chosen reference levels of the covariates. The indirect effects for smoking and their standard errors were obtained using proc nlmixed in SAS. For ease of parameter estimation, the models regarding smoking were adjusted for geographical location (Finland, Australia, Midwest, Bogalusa) instead of cohort.

Interaction

For each analysis, we tested the interaction term of childhood and adulthood risk factor in an additional model. Upon significant interaction, we would have proceeded to decompose the total effects into direct effects, mediated effects, interactive effects and effects due to both mediation and interaction. However, for each risk factor, the p-value of the interaction term was >0.05 for majority of the imputations, just not justifying further exploration.

Identifiability assumptions

The mediation analysis approach relies on set of assumptions that are generally untestable from the empirical data. These identifiability assumptions relate to having controlled for potential confounders.[6] The assumptions in the analyses presented here rely on the following identifiability conditions on confounding:

(i) There is no unmeasured confounding in the mediator-response relationship
(ii) There is no unmeasured confounding in the exposure-mediator relationship
(iii) There is no unmeasured confounding in the exposure-mediator relationship
(iv) The mediator-response confounders are not affected by the exposure.

© 2024 Kartiosuo N et al. *JAMA Network Open.*

B.2 Bayesian relevant life course model

To explore the relative importance of childhood risk factor exposures during different stages during the life-course and identify which life-course hypotheses are supported by our data, we estimated the relative contributions of each risk factor to the CVD event during different stages of life using Bayesian relevant life-course model (BRLM).[9]

In summary, BRLM aims to estimate the relative importance of exposure to the risk factors at different life-course periods. To this end, the model estimates weights based on the data and any given priors for the exposures during different stages of the life-course. The weights are allowed to take value between 0 and 1 so that the sum of the weights is 1. The relevant life-course exposure is then built as a weighted sum based on the estimated weights as follows:

$$l_i = \sum_{j=1}^{k} w_j x_j, \qquad (4)$$

Where l_i is the relevant life-course exposure, x_j is the exposure level at time j, w_j is the estimated weight for exposure level at time j and k is the number of time periods investigated.

The estimated weights may be interpreted to support different life-course hypotheses. Madathil et al present different types of potential hypotheses that the weights might support.[9] For example, if all periods have equal weight, the hypothesis supported the most is pure accumulation model. In contrast, if one period during the life-course is estimated to have a weight of one and the others have estimated weight of zero, critical period hypothesis is supported. Lastly, if multiple periods seem to be of importance, but their weights are not equal, sensitive period hypothesis may be supported.

We investigate the relative importance of the individual risk factors and the risk score measure at a particular period with $k = 2$ life periods (childhood, ages 3-19; adulthood, age 20 until the last measurement before event or censoring) and $k = 3$ life periods (early childhood, ages 3-11; later adolescence, ages 12-19; early adulthood, ages 20-29) life periods. The first choice corresponds to the "exposure" and "mediator" in our mediation analysis, whereas the latter division, in the absence of more accurate measures, represents childhood as pre-pubertal and post-pubertal life periods. We used the z-scores described previously as risk factor measures.[1]

In these rather exploratory analyses, we only investigated the odds of developing CVD, excluding the other than CVD-related deaths. We relied on the binomial model, and modelled the probability of event, p, based on a logistic function

$$\text{logit}(p(y = 1)) = (\beta_0 + \delta l x_i + \boldsymbol{\alpha C}),$$

where l_i is as described in Equation (4), $\boldsymbol{\alpha}$ is the vector of coefficients for the covariates $\boldsymbol{C}$ and β_0 is the intercept. The coefficient of the life-course effect, δ, describes the overall effect of the relevant life-course exposure on the response (i.e., the weighted sum of Equation (4)). Our set of covariates $\boldsymbol{C}$ included indicator of male sex, indicator of Black race, dummy variables indicating the geographic location of the participant (Australia, Finland, Midwest or Bogalusa) and z-scored average year of childhood examination to account for secular trends in the risk factors. Of note, the calendar year was standardised and the cohort re-classified into geographic location in order to obtain better convergence of the models.

We used non-informative prior distributions (i.e., Dirichlet(1,1,1) or Dirichlet(1,1)) for the weights of the risk factors in order to identify the hypotheses that are strongly supported by the data. For parameters $\beta_0, \delta, \alpha_1, \ldots, \alpha_6$ we used a weakly informative Cauchy(0, 2.5) distribution as the prior.

The model was fitted separately for each imputation. For each imputation, we calculated the posterior distribution means for the weights and the life-course coefficient δ with corresponding credible intervals. We here report the averages over the 10 imputations for these statistics and interpret then in terms of the life-course hypotheses that they potentially support.

© 2024 Kartiosuo N et al. *JAMA Network Open.*

eTable 1. The Z-scored risk factors by event status (mean (SD)) averaged over the 10 imputations.

Childhood risk factor	All (n=10634)	Non-event (n=10113.1)	CVD event (fatal or non-fatal, n=520.9)
**Z score for BMI (mean, sd)**	-0.16 (0.83)	-0.18 (0.81)	0.23 (1.06)
**Z score for SBP**	0.18 (0.92)	0.18 (0.91)	0.36 (1.07)
**Z score for TC**	0.30 (1.08)	0.30 (1.08)	0.21 (0.98)
Other cohorts than YFS	*0.00 (0.92)*	*-0.01 (0.92)*	*0.10 (0.89)*
YFS	*1.13 (1.04)*	*1.13 (1.04)*	*1.23 (1.13)*
**Z score for logTG**	0.02 (0.84)	0.00 (0.84)	0.31 (0.85)
**Z score for LDL-C**	0.20 (1.04)	0.20 (1.05)	0.15 (1.00)
Other cohorts than YFS	*-0.07 (0.91)*	*-0.07 (0.91)*	*0.04 (0.94)*
YFS	*0.92 (1.04)*	*0.91 (1.04)*	*1.16 (1.08)*
**Childhood risk score**	0.22 (0.47)	0.21 (0.46)	0.43 (0.54)
**Adulthood risk factor**			
**Z score for BMI**	-0.05 (0.93)	-0.07 (0.92)	0.37 (1.14)
**Z score for SBP**	0.05 (0.92)	0.03 (0.91)	0.35 (1.03)
**Z score for TC**	0.00 (0.93)	0.00 (0.92)	0.24 (1.03)
Other cohorts than YFS	*-0.07 (0.91)*	*-0.09 (0.93)*	*0.19 (1.00)*
YFS	*0.23 (0.88)*	*0.23 (0.87)*	*0.73 (1.04)*
**Z score for logTG**	-0.04 (0.92)	-0.06 (0.91)	0.40 (1.00)
**Z score for LDL-C**	-0.02 (0.93)	-0.03 (0.92)	0.25 (1.02)
Other cohorts than YFS	*-0.09 (0.94)*	*-0.11 (0.93)*	*0.20 (1.00)*
YFS	*0.19 (0.88)*	*0.18 (0.87)*	*0.71 (1.10)*
**Adulthood risk score**	0.12 (0.53)	0.10 (0.52)	0.49 (0.59)

Abbreviations: BMI = body mass index; SBP = systolic blood pressure; TC = total cholesterol; TG = triglycerides; LDL-C = LDL-cholesterol; YFS = Young Finns Study

C.1 Path-specific coefficients from the main mediation analyses

eTable 2. The path-specific coefficients for the indirect effects. Parameter β corresponds to the effect of childhood risk factor on the adulthood risk factor (mediator) and is based on a linear model for other risk factors than smoking and on logistic regression for smoking. Parameter γ_2 to the effect of the adulthood risk factor on CVD on the RR-scale.

Risk factor	β (95% CI)	γ_2 (95% CI)
**Score**	0.55 [0.53; 0.58]	2.63 [2.21; 2.13]
**Smoking***	1.90 [1.84; 1.96]	2.09 [1.61; 2.71]
**BMI**	0.77 [0.75; 0.78]	1.25 [1.12; 1.40]
**logTG**	0.37 [0.35; 0.39]	1.52 [1.38; 1.67]
**LDL-C**	0.45 [0.43; 0.47]	1.26 [1.12; 1.41]
**TC**	0.45 [0.42; 0.47]	1.33 [1.19 1.49]
**SBP**	0.37 [0.35; 0.39]	1.45 [1.31; 1.60]

Abbreviations: SBP = systolic blood pressure; TC = total cholesterol; TG = triglycerides; LDL-C = LDL-cholesterol;; RR=risk ratio; CI=confidence interval. *For smoking, the β coefficient is based on a logit-model and is here reported on the OR scale. For the continuous, variables, the coefficient is based on a linear model.

© 2024 Kartiosuo N et al. *JAMA Network Open.*

C.2 Mediation analyses for fatal events

The principal reason for examining associations for fatal events separately to those for all CVD events, is that follow up for death was available for virtually all participants who entered the study in childhood. The complete follow up for deaths removes the possibility of bias due to loss to follow up, which we cannot exclude when examining risk factor associations for 'all events'. When we compare the associations for fatal events with 'All' events we see, reassuringly, that the total, direct and indirect effects estimated are similar for both Fatal and All events

eTable 3. Risk ratios for the total, direct and indirect effects of childhood risk factors on the fatal cardiovascular events.

Risk factor	Total Effect (RR; 95% CI)	Direct Effect (RR; 95% CI)	Indirect Effect (RR; 95% CI)
**Score ****	2.74 [1.91; 3.92]	1.26 [0.82; 1.96]	1.97 [1.60; 2.43]
**BMI**	1.53 [1.31; 1.79]	1.30 [1.01; 1.67]	1.17 [0.98; 1.39]
**logTG**	1.44 [1.14; 1.83]	1.16 [0.89; 1.51]	1.18 [1.09; 1.27]
**SBP**	1.40 [1.13; 1.74]	1.06 [0.84; 1.35]	1.25 [1.16; 1.35]
**Smoking***	1.32 [0.87; 1.99]	0.78 [0.46; 1.33]	1.74 [1.24; 2.45]
**LDL-C**	1.30 [1.04; 1.61]	1.24 [0.94; 1.60]	1.05 [0.94; 1.17]
**TC**	1.29 [1.04; 1.61]	1.16 [0.88; 1.52]	1.08 [0.97; 1.21]

Abbreviations: BMI = body mass index; SBP = systolic blood pressure; TC = total cholesterol; TG = triglycerides; LDL-C = LDL-cholesterol; RR=risk ratio; CI=confidence interval.
* the effect of childhood risk factor on adulthood risk factor was calculated using logistic regression; ** the score includes BMI, SBP, TC, log TG and smoking.

C.3 Mediation analyses stratified by sex

In the mediation analyses stratified by sex, the effects of BMI, TC and LDL-C are nearly the same for males and females, and the effects of logTG are also quite similar between the sexes. However, in males, the direct effect of SBP is substantially larger than in females (1.19 [1.03; 1.37 for males; 0.98 [0.82; 1.16] for females). Smoking appears to have a larger indirect effect in females compared to males. This stems from larger effect of adulthood smoking in the models adjusted for childhood smoking in females (data not shown), which is in line with some previous reports[5].

eTable 4. Risk ratios for the total, direct and indirect effects of childhood risk factors on the fatal and non-fatal cardiovascular events stratified by sex.

	Males			Females		
**Risk factor**	**TE (RR; 95% CI)**	**DE (RR; 95% CI)**	**IE (RR; 95% CI)**	**TE (RR; 95% CI)**	**DE (RR; 95% CI)**	**IE (RR; 95% CI)**
**Score****	2.39 [1.90; 3.00]	1.39 [1.06; 1.83]	1.62 [1.41; 1.87]	2.43 [1.87; 3.14]	1.27 [0.93; 1.73]	1.77 [1.54; 2.03]
**Smoking***	1.33 [1.03; 1.72]	0.91 [0.66; 1.27]	1.47 [1.21; 1.79]	1.61 [1.19; 2.17]	0.93 [0.63; 1.37]	1.79 [1.35; 2.38]
**BMI**	1.40 [1.26; 1.57]	1.19 [1.00; 1.42]	1.17 [1.03; 1.34]	1.39 [1.22; 1.60]	1.19 [0.99; 1.43]	1.18 [1.04; 1.32]
**logTG**	1.33 [1.14; 1.54]	1.07 [0.91; 1.27]	1.17 [1.11; 1.23]	1.41 [1.20; 1.66]	1.13 [0.94; 1.35]	1.15 [1.11; 1.23]
**LDL-C**	1.34 [1.17; 1.53]	1.18 [1.00; 1.40]	1.11 [1.03; 1.19]	1.31 [1.10; 1.55]	1.14 [0.93; 1.41]	1.11 [1.02; 1.20]
**TC**	1.29 [1.13; 1.46]	1.08 [0.92; 1.27]	1.14 [1.06; 1.22]	1.27 [1.08; 1.51]	1.06 [0.86; 1.31]	1.14 [1.06; 1.23]
**SBP**	1.34 [1.18; 1.52]	1.19 [1.04; 1.37]	1.11 [1.06; 1.17]	1.18 [1.01; 1.39]	0.98 [0.82; 1.16]	1.18 [1.12; 1.26]

Abbreviations: BMI = body mass index; SBP = systolic blood pressure; TC = total cholesterol; TG = triglycerides; LDL-C = LDL-cholesterol; TE=total effect; DE =Direct effect; IE =indirect effect; RR=risk ratio; CI=confidence interval.* the effect of childhood risk factor on adulthood risk factor was calculated using logistic regression;
** the score includes BMI, SBP, TC, log TG and smoking.

C.4 Mediation analyses stratified by number of childhood visits

To gain insights on whether the effects are different for those who only had one measurement time during childhood compared to those who were followed up repeatedly in childhood, we stratified the analysis sample based on the number of measurement times (one vs. multiple measurements).

The effects within the participants with multiple childhood measurements seem to be larger and stronger for nearly all analyses compared to those with only one childhood visit. It is of note that those with one measurement may have slightly lower risk factor levels (data not shown). In addition, these participants are older at the time of event or

© 2024 Kartiosuo N et al. *JAMA Network Open.*

censoring than those who have been measured multiple times. Of note, cohort design affects the number of measurement times as well. For example, all CDAH participants and most Princeton participants only have one measurement in childhood, while in the remaining cohorts, the majority of participants have multiple measurements.

eTable 5. Risk ratios for the total, direct and indirect effects of childhood risk factors on the fatal and non-fatal cardiovascular events stratified by number of visits in childhood.

	One childhood visit			Multiple childhood visits		
**Risk factor**	**TE (RR; 95% CI)**	**DE (RR; 95% CI)**	**IE (RR; 95% CI)**	**TE (RR; 95% CI)**	**DE (RR; 95% CI)**	**IE (RR; 95% CI)**
**Score ****	1.86 [1.40; 2.47]	1.20 [0.87; 1.64]	1.41 [1.27; 1.56]	2.96 [2.33; 3.75]	1.38 [1.04; 1.85]	2.04 [1.71; 2.42]
**BMI**	1.32 [1.14; 1.54]	1.13 [0.93; 1.37]	1.17 [1.05; 1.31]	1.46 [1.31; 1.62]	1.23 [1.04; 1.45]	1.18 [1.04; 1.34]
**logTG**	1.17 [0.99; 1.39]	1.00 [0.84; 1.20]	1.10 [1.06; 1.15]	1.58 [1.35; 1.85]	1.21 [1.02; 1.43]	1.24 [1.16; 1.32]
**LDL-C**	1.22 [1.01; 1.46]	1.15 [0.94; 1.41]	1.05 [0.99; 1.11]	1.40 [1.23; 1.60]	1.16 [0.98; 1.38]	1.17 [1.08; 1.28]
**TC**	1.21 [1.02; 1.44]	1.09 [0.90; 1.33]	1.06 [1.02; 1.12]	1.33 [1.16; 1.52]	1.04 [0.87; 1.24]	1.22 [1.11; 1.34]
**SBP**	1.17 [1.01; 1.35]	1.08 [0.93; 1.25]	1.06 [1.03; 1.09]	1.41 [1.22; 1.64]	1.13 [0.96; 1.33]	1.26 [1.16; 1.37]

Abbreviations: BMI = body mass index; SBP = systolic blood pressure; TC = total cholesterol; TG = triglycerides; LDL-C = LDL-cholesterol; TE=total effect; DE =Direct effect; IE =indirect effect; RR=risk ratio; CI=confidence interval.* the score includes BMI, SBP, TC, log TG and smoking.

C.5 Mediation analyses adjusted for BMI

As childhood BMI can be thought to be a causal antecedent of the lipid measures and blood pressure in childhood, we took an additional sensitivity analysis adjusting for childhood and adulthood BMI. Similarly to the main analyses, we used the subject-specific average standardised BMI measures in these analyses. The DAG in Supplementary Figure 2 describes the assumed causal pathways.

eFigure 2. Hypothesised causal pathways of childhood SBP and lipids on CVDs, when BMI is considered antecedent of the risk factors.

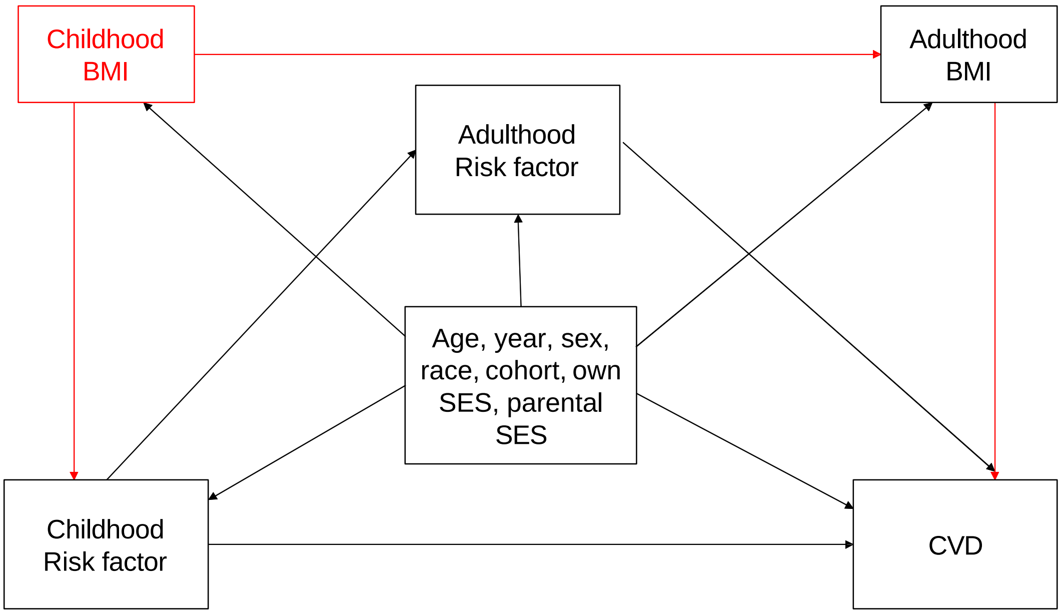

© 2024 Kartiosuo N et al. *JAMA Network Open.*

eTable 6. Risk ratios for the total, direct and indirect effects for the biological risk factors when adjusting for the BMI measurements.

Risk factor	Total Effect (RR; 95% CI)	Direct Effect (RR; 95% CI)	Indirect Effect (RR; 95% CI)
**LDL-C**	1.25 [1.11; 1.40]	1.12 [0.95; 1.29]	1.09 [1.04; 1.15]
**logTG**	1.24 [1.11; 1.40]	1.03 [0.91; 1.17]	1.14 [1.10; 1.18]
**TC**	1.23 [1.11; 1.37]	1.06 [0.93; 1.21]	1.11 [1.06; 1.17]
**SBP**	1.14 [1.03; 1.27]	1.01 [0.91; 1.14]	1.11 [1.07; 1.16]

Abbreviations: SBP = systolic blood pressure; TC = total cholesterol; TG = triglycerides; LDL-C = LDL-cholesterol; TE=total effect; DE =Direct effect; IE =indirect effect; RR=risk ratio; CI=confidence interval.

© 2024 Kartiosuo N et al. *JAMA Network Open.*

eTable 7. Risk ratios for the total, direct and indirect effects treating the non-CVD deaths as nonevents

Risk factor	Total Effect (RR; 95% CI)	Direct Effect (RR; 95% CI)	Indirect Effect (RR; 95% CI)
**Score ****	2.21 [1.87; 2.62]	1.26 [1.03; 1.54]	1.64 [1.49; 1.82]
**BMI**	1.35 [1.25; 1.46]	1.16 [1.03; 1.31]	1.15 [1.06; 1.25]
**logTG**	1.33 [1.19; 1.49]	1.08 [0.95; 1.22]	1.16 [1.12; 1.20]
**SBP**	1.25 [1.13; 1.38]	1.09 [0.98; 1.21]	1.14 [1.10; 1.18]
**Smoking***	1.40 [1.14; 1.70]	0.91 [0.71; 1.18]	1.49 [1.28; 1.73]
**LDL-C**	1.32 [1.18; 1.47]	1.15 [1.00; 1.33]	1.11 [1.06; 1.17]
**TC**	1.28 [1.15; 1.42]	1.08 [0.95; 1.23]	1.13 [1.08; 1.19]

eTable 8. Risk ratios for the total, direct and indirect effects for total and LDL-C without Finnish participants

Risk factor	Total Effect (RR; 95% CI)	Direct Effect (RR; 95% CI)	Indirect Effect (RR; 95% CI)
**LDL-C**	1.33 [1.21; 1.47]	1.19 [1.06; 1.34]	1.09 [1.04; 1.13]
**TC**	1.30 [1.17; 1.44]	1.10 [0.98; 1.25]	1.11 [1.06; 1.16]

© 2024 Kartiosuo N et al. *JAMA Network Open.*

eReferences

1. D. R. Jacobs J, Woo JG, Sinaiko AR, et al. Childhood Cardiovascular Risk Factors and Adult Cardiovascular Events. *N Engl J Med*. 2022;386(20):1877. doi:10.1056/NEJMoa2109191

2. Chen X, Wang Y, Appel LJ, Mi J. Impacts of measurement protocols on blood pressure tracking from childhood into adulthood: a metaregression analysis. *Hypertens Dallas Tex 1979*. 2008;51(3):642-649. doi:10.1161/HYPERTENSIONAHA.107.102145

3. Friedewald WT, Levy RI, Fredrickson DS. Estimation of the concentration of low-density lipoprotein cholesterol in plasma, without use of the preparative ultracentrifuge. *Clin Chem*. 1972;18(6):499-502.

4. Hu T, Gall SL, Widome R, et al. Childhood/Adolescent Smoking and Adult Smoking and Cessation: The International Childhood Cardiovascular Cohort (i3C) Consortium. *J Am Heart Assoc Cardiovasc Cerebrovasc Dis*. 2020;9(7). doi:10.1161/JAHA.119.014381

5. Sinaiko AR, Jacobs DR, Woo JG, et al. The International Childhood Cardiovascular Cohort (i3C) consortium outcomes study of childhood cardiovascular risk factors and adult cardiovascular morbidity and mortality: Design and recruitment. *Contemp Clin Trials*. 2018;69:55-64. doi:10.1016/j.cct.2018.04.009

6. Valeri L, VanderWeele TJ. Mediation analysis allowing for exposure-mediator interactions and causal interpretation: theoretical assumptions and implementation with SAS and SPSS macros. *Psychol Methods*. 2013;18(2):137-150. doi:10.1037/a0031034

7. VanderWeele TJ. Mediation Analysis: A Practitioner's Guide. *Annu Rev Public Health*. 2016;37(1):17-32. doi:10.1146/annurev-publhealth-032315-021402

8. Yang W, Jepson C, Xie D, et al. Statistical Methods for Recurrent Event Analysis in Cohort Studies of CKD. *Clin J Am Soc Nephrol CJASN*. 2017;12(12):2066-2073. doi:10.2215/CJN.12841216

9. Madathil S, Joseph L, Hardy R, Rousseau MC, Nicolau B. A Bayesian approach to investigate life course hypotheses involving continuous exposures. *Int J Epidemiol*. 2018;47(5):1623-1635. doi:10.1093/ije/dyy107

© 2024 Kartiosuo N et al. *JAMA Network Open.*
