# Supplementary material for: Cardiovascular Risk Factors in Childhood and Adulthood and Cardiovascular Disease in Middle Age
Source: JAMA Netw Open. 2024 Jun 24;7(6):e2418148. doi: 10.1001/jamanetworkopen.2024.18148 (PMC11197443; doi:10.1001/jamanetworkopen.2024.18148)
Supplement: Supplement 2. — Data Sharing Statement [file jamanetwopen-e2418148-s002.pdf]

Data Sharing Statement

Kartiosuo. Cardiovascular Risk Factors in Childhood and Adulthood and Cardiovascular Disease in Middle Age. *JAMA Netw Open*. Published June 24, 2024.
doi:10.1001/jamanetworkopen.2024.18148

Data
Data available: No

Additional Information
Explanation for why data not available: Data collected for this study outside EU, including deidentified individual participant data, complete data dictionaries, and other related documents, are available for reasonable request from the chair of the i3C consortium (Terry Dwyer, terence.dwyer@wrh.ox.ac.uk). Data collected within EU (The YFS dataset) comprises health related participant data and their use is therefore restricted under the regulations on professional secrecy (Act on the Openness of Government Activities, 612/1999) and on sensitive personal data (Personal Data Act, 523/1999, implementing the EU data protection directive 95/46/EC). Due to these legal restrictions, the data from YFS study can not be made publicly available. However, data access may be permitted on a case-by-case basis upon request only. Data sharing outside the group is done in collaboration with the YFS group and requires a data-sharing agreement. Investigators can submit an expression of interest to the coordinator of the YFS (Olli Raitakari, olli.raitakari@utu.fi).
